# Beyond adaptive immunity: Functional diversity of the type III-A CRISPR-Cas system in *Mycobacterium tuberculosis*

**DOI:** 10.1016/j.cellin.2026.100342

**Published:** 2026-06-15

**Authors:** Wenjing Yu, Xindi Huang, Yangbo Hu, Shiyun Chen

**Affiliations:** State Key Laboratory of Virology and Biosafety, Wuhan Institute of Virology, Chinese Academy of Sciences, Wuhan, 430071, China

**Keywords:** *Mycobacterium tuberculosis*, Type III-A CRISPR-Cas system, Immunity, Functional diversity

## Abstract

CRISPR-Cas systems are best known as prokaryotic adaptive immune pathways that defend against invading genetic elements. *Mycobacterium tuberculosis* (*Mtb*) harbors a type III-A CRISPR-Cas system that is structurally conserved yet exhibits little evidence of ongoing spacer acquisition. Nevertheless, its interference machinery remains functional, and increasing evidence suggests that this system has evolved roles beyond canonical adaptive immunity. Accumulating studies indicate that this system is deeply integrated into cellular regulatory networks by governing stress responses, metabolic adaptation, and host-pathogen interactions. Mechanistically, the *Mtb* type III-A CRISPR-Cas system operates through transcription-dependent target recognition and cyclic oligoadenylate (cOA)-mediated signal amplification, in which the ancillary ribonuclease Csm6 serves as a key effector. Functionally, CRISPR-associated proteins influence antibiotic susceptibility, oxidative stress resistance and host immune responses, and may even act as secreted immunomodulatory factors. In this review, we summarize current understanding of the genomic organization, regulatory mechanisms, and non-canonical functions of the *Mtb* type III-A CRISPR-Cas system, with particular emphasis on its emerging roles in stress adaptation and host immune regulation.

## 1 Introduction

Clustered regularly interspaced short palindromic repeats (CRISPR) and CRISPR-associated proteins (Cas) constitute a key adaptive immune mechanism in prokaryotes, providing sequence-specific protection against invading viral and plasmid nucleic acids (Barrangou and Horvath, 2017). Beyond this canonical role, CRISPR-Cas systems are increasingly recognized as regulators of diverse bacterial physiological processes (Mohanraju et al., 2022), including virulence (Li et al., 2025; Ratner et al., 2019; Sampson et al., 2013; Sharma et al., 2024), biofilm formation (Guo et al., 2025; Tang et al., 2019), quorum sensing (Li et al., 2016; Zegans et al., 2009), and antibiotic resistance (Mo et al., 2021; Wei et al., 2025). These non-canonical functions often involve the regulation of endogenous gene expression. For example, mechanistic studies in *Francisella novicida* demonstrated that the scaRNA-tracrRNA-FnCas9 complex represses endogenous targets through transcriptional interference, providing a molecular basis for CRISPR-Cas-mediated regulation of bacterial virulence and host immune responses (Ratner et al., 2019). These and other recent mechanistic studies have substantially expanded our understanding of CRISPR-Cas systems as multifunctional regulators of bacterial physiology beyond their established role in adaptive immunity.

Based on effector complex composition and *cas* gene architecture, CRISPR-Cas systems are broadly classified into two major classes encompassing seven types. Class 1 systems (types I, III, IV and VII) employ multi-subunit CRISPR RNA (crRNA)-effector complexes to mediate RNA and/or DNA degradation, whereas Class 2 systems (types II, V, and VI) rely on a single multidomain effector protein to carry out interference (Altae-Tran et al., 2023; Makarova et al., 2025). Among these, the type III CRISPR-Cas system, which belongs to Class 1, is of particular interest owing to its unique transcription-dependent interference mechanism. Unlike DNA-targeting CRISPR-Cas systems, type III CRISPR-Cas systems recognize phage-derived transcripts through crRNA-guided Cas10-Csm effector complexes (Samai et al., 2015; Tamulaitis et al., 2017). Upon target RNA binding, the Csm3 cleaves the RNA (Staals et al., 2013, 2014). Concurrently, target RNA recognition activates the HD nuclease domain of Cas10, leading to the degradation of actively transcribed phage DNA (Kazlauskiene et al., 2017). In parallel, the Palm domain of Cas10 synthesizes cyclic oligoadenylates (cOAs), which serve as second messengers to activate auxiliary nucleases such as Csm6 and Csx1, triggering a broad RNA degradation response that further restricts phage replication (Niewoehner et al., 2017). Through the coordinated activities of RNA cleavage, transcription-coupled DNA degradation, and cOA-mediated signaling, type III CRISPR-Cas systems provide robust antiviral defense while minimizing self-targeting (van Beljouw et al., 2023).

*Mycobacterium tuberculosis* (*Mtb*), the leading cause of death from a single infectious agent worldwide, remains incompletely understood in terms of the mechanisms underlying latent infection and the emergence of drug resistance. As a prototypical intracellular pathogen, *Mtb* resides within host cells and forms granulomas, where it is continuously exposed to multiple environmental stresses, e.g., nutrient limitation (Devlin et al., 2025; Yang et al., 2014), oxidative stress (Xu et al., 2025), hypoxia (McCaffrey et al., 2026), and immune pressures (Pepper-Tunick et al., 2026; Saraiva et al., 2026). Recent studies have shown that CRISPR-Cas systems respond to diverse cellular stress signals, such as envelope perturbation, heat shock, and oxidative stress (Devi et al., 2022). Notably, the *Mtb* genome harbors a single, structurally intact type III-A CRISPR-Cas system and lacks other known CRISPR-Cas types (He et al., 2012; Zhang et al., 2024). Although this system retains the core components required for anti-phage immunity, accumulating evidence suggests that its functional repertoire extends far beyond classical immune defense, having evolved to fulfill more complex biological roles (Hamdi et al., 2023).

In this review, we summarize recent advances in understanding the *Mtb* type III-A CRISPR-Cas system, covering its genomic organization, evolutionary features, and defense activities against phages and plasmids. We then discuss the regulation of the *cas* genes expression, the molecular mechanisms of target recognition and signal amplification, and the emerging non-canonical functions of this system in virulence, host interaction, and stress adaptation.

## 2 CRISPR organization and evolutionary features

CRISPR sequences in mycobacteria were first identified in 1991 and, owing to the polymorphism of their repeat and spacer sequences, have been widely used for mycobacterial spoligotyping analyses (Groenen et al., 1993; Hermans et al., 1991). Subsequent genomic studies revealed that nearly all members of the *Mtb* complex (MTBC) encode a conserved type III-A CRISPR-Cas system centered on the Cas10-Csm effector complex. The CRISPR-Cas locus typically comprises two major CRISPR arrays (CRISPR1 and CRISPR2), separated by insertion sequences such as IS6110, and accompanied by a full complement of *cas* genes, including *cas1*, *cas2*, *cas6*, *cas10*, and *csm2-csm5* (He et al., 2012). This genomic architecture suggests that the system retains the components required for adaptive immunity.

However, evolutionary analyses have revealed an atypical pattern. Spacer sequences within *Mtb* CRISPR arrays bear no detectable sequence homology to known mycobacteriophages, and large-scale genomic studies of clinical isolates have failed to identify evidence of ongoing spacer acquisition (Freidlin et al., 2017; He et al., 2012; Refregier et al., 2020; Supply et al., 2013). Instead, CRISPR loci have evolved primarily through structural variation, including spacer loss, repeat polymorphisms, and IS-mediated rearrangements (Refregier et al., 2020). And mutations in *cas1* are also frequently observed in clinical isolates (Wei et al., 2019a). These observations indicate that the adaptation phase of the *Mtb* type III-A CRISPR-Cas system may be largely inactive in modern MTBC lineages. Together, these features define a system characterized by structural conservation but functional decay in spacer acquisition, suggesting that selective pressures may have shifted its role away from classical adaptive immunity.

## 3 Defense activity against phages and plasmids

Despite these evolutionary signatures of functional decay in spacer acquisition (Freidlin et al., 2017; He et al., 2012; Refregier et al., 2020; Supply et al., 2013), experimental evidence indicates that the *Mtb* type III-A CRISPR-Cas system retains the capacity to mediate defense against foreign nucleic acids. The interference activity of this system has been experimentally demonstrated under appropriate conditions. In heterologous hosts such as *Escherichia coli*, introduction of cognate target sequences into plasmids markedly reduced transformation efficiency, indicating that the *Mtb* type III-A CRISPR-Cas system is capable of mediating effective anti-plasmid interference when suitable targets are present (Grüschow et al., 2019). Similarly, in *Mycolicibacterium smegmatis* (*Msm*), overexpression of the *Mtb* type III-A CRISPR-Cas system conferred partial protection against TM4 infection when target sequences derived from the TM4 phage genome were incorporated into the experimental assay, thereby supporting its anti-phage activity (Yu et al., 2026). Because CRISPR interference is sequence-specific and depends on target transcription, its efficacy is shaped not only by target sequence complementarity but also by the transcriptional orientation and expression level of the target locus. Therefore, the absence of detectable resistance under certain conditions should not simply be interpreted as a loss of immune function. Collectively, these findings indicate that the *Mtb* type III-A CRISPR-Cas system remains a functional interference system capable of anti-plasmid and anti-phage activity under defined conditions, albeit with limited evidence of ongoing spacer acquisition.

## 4 Mechanism of target recognition and signal amplification

The type III-A CRISPR-Cas system in *Mtb* employs a transcription-dependent mechanism for target recognition, which is central to canonical interference (Samai et al., 2015; Tamulaitis et al., 2017). Like other type III-A systems, precursor CRISPR RNAs (pre-crRNAs) are processed into mature crRNAs that assemble with Csm proteins to form the Cas10-Csm effector complex (Staals et al., 2014). Guided by crRNAs, the complex binds nascent RNA transcripts, allowing discrimination of actively transcribed foreign elements. Upon target engagement, the Csm3 subunit mediates sequence-specific RNA cleavage, while the Cas10 Palm domain synthesizes cOAs that activate the auxiliary RNase Csm6, amplifying the immune response (Grüschow et al., 2019; Kazlauskiene et al., 2017; Niewoehner et al., 2017; Yu et al., 2026).

**Fig. 1:**
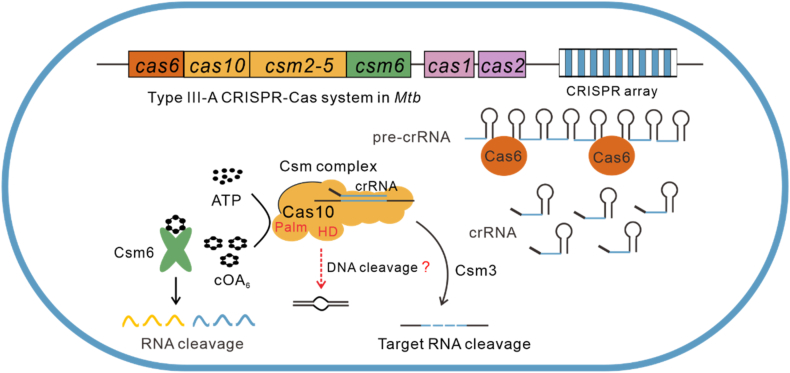
**Schematic representation of the organization and immunity mechanism of the type III-A CRISPR-Cas system in *Mtb*.** The *Mtb* type III-A CRISPR-Cas locus consists of *cas6*, *cas10*, *csm2-csm5*, *csm6*, *cas1*, *cas2*, and the CRISPR array. The CRISPR array is first transcribed into pre-crRNA, which is then processed by Cas6 into mature crRNA and assembled into the Cas10-Csm effector complex. Upon target transcript recognition, Csm3 mediates sequence-specific cleavage of target RNA, while the palm domain of Cas10 synthesizes cOA_6_, which activates the auxiliary RNase Csm6 and triggers nonspecific RNA degradation. The HD domain of Cas10 may contribute to DNA cleavage, although its precise role remains to be clarified.

The organization and immunity mechanism of the type III-A CRISPR-Cas system in *Mtb* are summarized in Fig. 1. Although the *Mtb* type III-A CRISPR-Cas system retains the conserved transcription-dependent immune framework, several mechanistic features appear to be specific to mycobacteria. In particular, *Mtb* Cas6 mediates metal-dependent pre-crRNA processing, generating mature crRNAs of defined lengths (Wei et al., 2019b). Functional studies have shown that *Mtb* Csm6 is preferentially activated by cOA_6_ rather than other cyclic oligoadenylates, and that the Cas10 Palm domain together with Csm6 constitutes the primary effector module, while the HD nuclease domain contributes minimally to interference (Grüschow et al., 2019; Yu et al., 2026). In addition, Orn-dependent degradation of nanoRNAs (2-5 nt oligoribonucleotides) and intracellular c-di-GMP signaling provide unique regulatory layers that modulate CRISPR-Cas activity in *Mtb* (Yu et al., 2026). Collectively, these adaptations highlight how the *Mtb* type III-A system has evolved distinct effector and regulatory strategies, enabling a finely tuned immune response within the intracellular environment, beyond the conserved mechanisms shared with other type III-A systems.

## 5 Expression regulation and integration into stress response networks

The *Mtb* type III-A CRISPR-Cas system appears to be linked to broader stress response networks. As the primary causative agent of tuberculosis, *Mtb* encounters diverse environmental stresses within the host, including acidic pH, reactive oxygen species (ROS), reactive nitrogen species (RNS), antibiotic exposure, and the hostile conditions of granulomatous lesions (Devlin et al., 2025; McCaffrey et al., 2026; Pepper-Tunick et al., 2026; Saraiva et al., 2026; Xu et al., 2025; Yang et al., 2014). Transcriptomic analyses have shown that *cas* gene expression is induced under diverse stress conditions, particularly during latent infection or dormancy (Baker et al., 2014; Ignatov et al., 2015; Rodriguez et al., 2014). Functional analyses further demonstrated that deletion of the type III-A CRISPR-Cas system in *Mtb* increases sensitivity to oxidative stress and impairs intracellular survival (Yu et al., 2026), thereby highlighting a role in stress adaptation.

At the molecular level, the expression of the type III-A CRISPR-Cas system in *Mtb* is modulated by multiple regulatory pathways. The degradation of nanoRNAs has emerged as a critical regulatory layer: deletion of *cnpB*, a gene encoding a cyclic di-AMP phosphodiesterase with nanoRNA-processing activity, led to pronounced upregulation of *cas* genes (Zhang et al., 2018). Similarly, our work demonstrated that the oligoribonuclease Orn modulates intracellular c-di-GMP levels, which in turn regulate *cas* promoter activity via the c-di-GMP-responsive transcription factor Rv3058 (Yu et al., 2026). In addition to these global regulatory inputs, fine-tuned control is exerted at the level of individual system components. The transcription factor Rv1776c (CasR) directly binds the *csm6* promoter and represses its expression, likely preventing excessive RNA degradation (Wei et al., 2023). Collectively, these findings demonstrate that the *Mtb* type III-A CRISPR-Cas system is embedded within a multilayered regulatory network that links stress sensing, second-messenger signaling, and transcriptional control.

## 6 Host interaction, virulence, and functional diversification

**Fig. 2:**
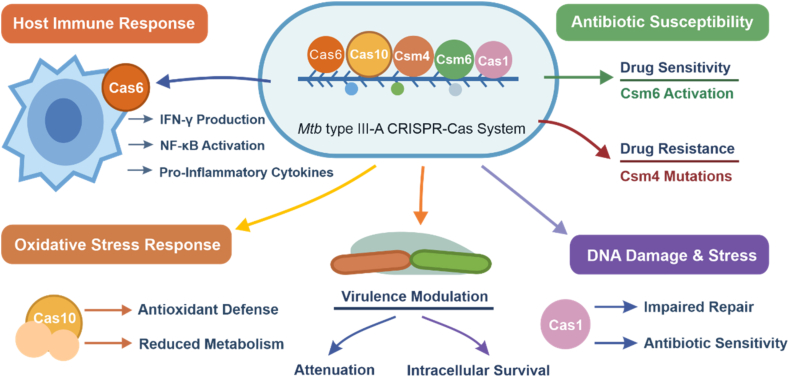
**Schematic illustration of the noncanonical functions of the *Mtb* type III-A CRISPR-Cas system.** In addition to immune defense, the *Mtb* type III-A CRISPR-Cas system is involved in host immune modulation, stress adaptation, antibiotic susceptibility, and virulence regulation. Cas6 can stimulate host inflammatory responses, whereas Csm4 and stress-induced activation of Csm6 enhances antibiotic sensitivity. Cas10 contributes to oxidative stress resistance and adaptation to antibiotic challenge, and Cas1 affects DNA damage repair and stress susceptibility. These observations suggest that the type III-A CRISPR-Cas system functions as a multifunctional regulatory module in mycobacterial physiology and pathogenesis.

Given the intracellular lifestyle of *Mtb*, exposure to bacteriophages during infection is likely limited (Russell et al., 2010). During long-term adaptation to the intracellular host environment, the type III-A CRISPR-Cas system appears to have acquired functions beyond immune defense, and some of these roles may persist even without a fully intact CRISPR locus (Refregier et al., 2020). These observations may suggest that the *Mtb* type III-A CRISPR-Cas system has evolved into a multifunctional module contributing to host interaction, bacterial physiology, and virulence. As outlined in Fig. 2, the *Mtb* type III-A CRISPR-Cas system encompasses multiple functions, including host immune modulation (Jiao et al., 2021), stress adaptation (Wei et al., 2019a), antibiotic susceptibility (Wei et al., 2025; Xu et al., 2024), and virulence regulation (Freidlin et al., 2017; Supply et al., 2013).

At the host-pathogen interface, one prominent noncanonical role of this system is the modulation of immune responses. Several Cas proteins have been detected in culture filtrates, suggesting secretion into the extracellular environment. Notably, Cas6 has been shown to induce IFN-γ production in human immune cells, activate NF-κB signaling, and stimulate pro-inflammatory cytokine release, indicating a direct role in modulating host immune responses (Jiao et al., 2021).

In addition to influencing host immunity, CRISPR-Cas activity appears to shape the response of *Mtb* to antimicrobial and environmental stress. Multiple observations support a link between CRISPR components and antibiotic susceptibility. While mutations in Cas proteins are associated with drug-resistance phenotypes (Xu et al., 2024), stress-induced activation of Csm6 appears to enhance drug sensitivity (Wei et al., 2025). Under environmental stress, bacteria accumulate high levels of nucleotide-derived signaling molecules that bind to Csm6 and stimulate its ssRNA ribonuclease activity. Csm6-mediated RNA degradation has been shown to increase susceptibility to multiple anti-tuberculosis drugs, likely by suppressing translation and repressing mycolic acid biosynthesis (Wei et al., 2025). Notably, in certain *Mtb* Beijing lineage strains with partial deletions of CRISPR operons, the CRISPR-Cas system still responds to stress, and Cas10 remains intact (Zhai et al., 2018). Supporting these strain-level observations, our recent work demonstrates that the *Mtb* type III-A CRISPR-Cas system participates in adaptation to oxidative stress and antibiotic challenge, a role that is mainly mediated by the Cas10 HD domain (Yu et al., 2026). Beyond the type III-A effector complex, in a distinct mechanism, Cas1 expression impairs DNA damage repair and increases susceptibility to multiple antibiotics and environmental stresses in *Msm* (Wei et al., 2019a). In addition, a K114N mutation in Rv2820c, which encodes the CRISPR-associated Csm4 subunit of the type III-A CRISPR-Cas effector complex, is highly enriched in capreomycin-resistant strains (Xu et al., 2024). Furthermore, this mutation has been associated with increased capreomycin tolerance, providing additional evidence for a potential link between CRISPR-associated factors and antibiotic resistance (Xu et al., 2024; Zhai et al., 2018).

Because immune modulation and antibiotic stress adaptation can influence bacterial persistence within the host, the potential contribution of the type III-A CRISPR-Cas system to *Mtb* pathogenicity has attracted increasing attention. However, direct evidence linking this system to virulence remains limited. Comparative genomic studies have identified substantial variation in CRISPR-Cas loci among tuberculosis-causing mycobacteria, including smooth tubercle bacilli strains that display reduced persistence and virulence compared with *Mtb* (Supply et al., 2013). In addition, structural polymorphisms within CRISPR loci have been reported among clinical isolates (Freidlin et al., 2017). While these observations suggest that CRISPR-Cas systems may be associated with strain-specific evolutionary adaptation, their direct contribution to *Mtb* virulence has not yet been established and warrants further investigation.

## 7 Conclusions and perspectives

The type III-A CRISPR-Cas system in *Mtb* represents an evolutionary intermediate between canonical adaptive immunity and regulatory complexity. While it retains key features of type III-A systems, including transcription-dependent target recognition, RNA cleavage, and cOA-mediated signaling (van Beljouw et al., 2023), several mechanistic nuances distinguish the *Mtb* system from other well-characterized type III-A systems. These include metal-dependent Cas6-mediated crRNA processing (Wei et al., 2019b), preferential cOA_6_-dependent activation of Csm6 (Grüschow et al., 2019), and regulation through the Orn-c-di-GMP signaling axis (Yu et al., 2026).

Beyond canonical immunity, emerging evidence indicates that the *Mtb* type III-A system contributes to additional adaptive functions. These include oxidative stress tolerance (Yu et al., 2026), antibiotic tolerance (Wei et al., 2019a), and host-pathogen interactions (Jiao et al., 2021), indicating that it may contribute to bacterial survival and persistence within the host environment. Notably, the c-di-GMP-responsive transcription factor Rv3058 has been shown to directly regulate CRISPR-Cas expression, linking the system to wider second-messenger and stress-response networks (Yu et al., 2026).

Despite these advances, the regulatory roles of the *Mtb* type III-A system remain incompletely understood. Key questions include the identification of endogenous regulatory targets, the molecular mechanisms underlying oxidative stress resistance and antibiotic tolerance, and the functional significance of the Cas10 HD domain. Future studies should also explore how environmental and host-derived signals modulate CRISPR-Cas activity during infection. Addressing these questions will not only deepen our understanding of *Mtb* physiology and pathogenesis but may also reveal novel targets for tuberculosis intervention.

## CRediT authorship contribution statement

**Wenjing Yu:** Writing – review & editing, Writing – original draft. **Xindi Huang:** Writing – review & editing. **Yangbo Hu:** Writing – review & editing. **Shiyun Chen:** Writing – review & editing, Writing – original draft, Funding acquisition.

## Declaration of competing interest

The authors declare that they have no known competing financial interests or personal relationships that could have appeared to influence the work reported in this paper.
